# Cultivating patient-centered care competence through a telemedicine-based course: An explorative study of undergraduate medical students’ self-reflective writing

**DOI:** 10.3389/fpubh.2023.1134496

**Published:** 2023-04-05

**Authors:** Ardi Findyartini, Chaina Hanum, Dewi Anggraeni Kusumoningrum, Azis Muhammad Putera, Retno Asti Werdhani, Oktavinda Safitry, Dina Muktiarti, Dewi Sumaryani Soemarko, Wismandari Wisnu

**Affiliations:** ^1^Department of Medical Education, Faculty of Medicine Universitas Indonesia, Jakarta, Indonesia; ^2^Medical Education Center, Indonesia Medical Education and Research Institute, Faculty of Medicine Universitas Indonesia, Jakarta, Indonesia; ^3^Clinical Clerkship - Undergraduate Medical Program, Faculty of Medicine Universitas Indonesia, Jakarta, Indonesia; ^4^Department of Community Medicine, Faculty of Medicine, Universitas Indonesia, Jakarta, Indonesia; ^5^Department of Forensic Medicine and Medicolegal Studies, Faculty of Medicine Universitas Indonesia, Cipto Mangunkusumo National Central Referral Hospital, Jakarta, Indonesia; ^6^Department of Child Health, Faculty of Medicine Universitas Indonesia, Cipto Mangunkusumo National Central Referral Hospital, Jakarta, Indonesia; ^7^Department of Internal Medicine, Faculty of Medicine Universitas Indonesia, Cipto Mangunkusumo National Central Referral Hospital, Jakarta, Indonesia

**Keywords:** patient-centered care (MeSH term), telemedicine, course, undergraduate (MeSH), self-reflective, explorative study

## Abstract

**Background:**

The COVID-19 pandemic has encouraged adaptations of learning methods in clinical clerkship. There have been limited reports on the merits of involving medical students in telemedicine. This study, therefore, aims to investigate students’ reflection on what they learned and identify the challenges and benefits of doctor-patient interaction through their experience in a telemedicine-based course.

**Methods:**

A 4 week telemedicine-based course for medical students to participate in telemonitoring of COVID-19 patients undergoing self-isolation was conducted. This is a qualitative study using an interpretive phenomenology design to investigate students’ self-reflection on their experiences in monitoring COVID-19 patients. Students were asked to reflect on their experience upon completion of the course through 750–1,000 words essays. A thematic analysis which considers units of meaning based on students’ experiences was completed.

**Results:**

Our study identified four main themes gathered from students’ experiences related to the telemedicine-based course: communication and education, professionalism and professional identity formation, system-based practice, and patient-centered care.

**Conclusion:**

The course was part of an integrative effort involving multiple parties to tackle the burden on the nation’s healthcare system during the pandemic. Telemedicine is part of future medical practice which supports the medical curriculum adaptability along with attempts to develop future-proof medical doctors through various clinical learning experiences.

## 1. Introduction

Self-isolation is one of the strategies to control the transmission of COVID-19, along with testing and tracing of COVID-19 cases (1–3). Self-isolation protocol includes staying at home and minimizing physical contact with other people, wearing masks, continuous self-monitoring of vital signs whenever possible, taking medications, and adjusting home ventilation. Due to the high surge of COVID-19 cases, health authorities in many countries encourage COVID-19 patients with mild or no symptoms to undergo self-isolation, to save hospital beds and ICUs for moderate to severe cases (4, 5) and to control disease transmission (6).

During the COVID-19 pandemic, telemedicine plays an important role in providing various health care services, especially in screening, assessing, and monitoring COVID-19 patients (7, 8). Telemedicine is the use of communication technology to provide healthcare services at a distance (9), enabling patients to receive medical care in a safe environment with a low risk of infection transmission. With telemedicine, the provider can obtain data related to a patient’s daily symptoms and their progression, assess the risk of exposure, and perform observational assessment, such as temperature, respiratory rate, and general appearance (10, 11).

The COVID-19 pandemic has had a huge impact on undergraduate medical students in the clinical phase. Medical schools worldwide had to withdraw their medical students from clinical rotations to ensure students’ safety (12, 13) and change the traditional learning process into a distant and online learning (14). This rearrangement resulted in the loss of essential learning opportunities (15, 16) and limiting students’ exposure to clinical case management and direct patient interactions (17). Many medical students reported anxiety and depression due to inadequate skills and competency attainment following suspension of hospital placements (13).

Several adaptations in clinical clerkship have been implemented to support student education during the pandemic. In the medical education context, telemedicine can be used as a means for engaging medical students in patient care, especially in times of crisis (18, 19). Telemedicine can be incorporated into the curriculum to facilitate students in developing competencies in patient care and to involve students in system-based practices (20). However, a study conducted by Franklin et al. (14) reported that some of the clinical students feel that the telemedicine program that was rapidly set up due to the COVID-19 was fragmented and somewhat to be less effective than in-person patient interactions.

Another concern with telemedicine practice is related to the patient-centered care framework, a framework aiming to enhance patient’s participation in keeping their health status and providing them with necessary support, often involving other healthcare professionals (21–23). Patient-centered care considers doctor-patient relationship and identification of the patient’s ideas, concerns, and expectations, which are expected to increase compliance. Good communication and rapport building between physicians and patients are pivotal to achieve this, but evidence showed that ensuring good communication to achieve patient-centered practice by utilizing telemedicine remained a problem (24).

There have been limited reports on the merits of involving medical students in patient care based on the telemedicine approach. This research, therefore, aims to explore students’ reflection on what they learned and identify the challenges and benefits of doctor-patient interaction through experience in a telemedicine-based course. With the COVID-19 pandemic in a country with limited resources as a context, this study highlights two research questions: 1) What did the medical students learn from their involvement in a telemedicine-based course? 2) What were the challenges and benefits of doctor-patient interaction through medical students’ experiences in the telemedicine-based course?

## 2. Methods

### 2.1. Context

Due to inadequacy of existing infrastructure in handling COVID-19 pandemic in Indonesia, monitored self-isolation became an intervention encouraged by the health authorities. (25, 26) The high number of COVID-19 cases had made people seek medical help to the hospital while its capacity was limited. It forced the health authorities to strengthen the monitoring program for COVID-19 patients with mild symptoms doing their home isolation. This was in accordance with the COVID-19 management standards where patients with mild symptoms need to be isolated and monitored at home. Patients undergoing self-isolation were encouraged to stay at home, monitor their vital signs if possible, and take medications given by the health authorities.

The Faculty of Medicine Universitas Indonesia (FMUI) has developed a four-week Self-Isolation Monitoring Module for 208 clinical students of Undergraduate Medical Program. This four-week course (from July–August 2021) aimed to enrich students’ experience in interacting with patients through safe clinical exposure as the clinical placement was halted temporarily due to the pandemic. Students had taken the clerkship oath and passed the basic medicine and surgery clinical rotations, thus had acquired the fundamental knowledge to monitor at least one COVID-19 patient undergoing self-isolation at home who showed mild or no symptoms. Monitoring was performed through telemedicine, i.e., video call, chat application, the COVID-19 Monitoring and Information Center (COMIC)® developed by Dr. Cipto Mangunkusumo Hospital (a teaching hospital affiliated to FMUI) and *Sistem Informasi Pelacakan* (SILACAK)®, COVID-19 information system developed by the Indonesian Ministry of Health. Each student interacted with 1–2 patients for several days during the self-isolation monitoring.

Prior to monitoring patients, students were given introductory lectures to enrich their knowledge to monitor COVID-19 patients. Throughout the course, students were divided into 29 groups and monitored the COVID-19 patients through telemedicine under tutor supervision. Each tutor was assigned for one group, consisting of 7–8 students. This course involved tutors from various departments in our medical school. Students were required to fill the monitoring sheet daily and discussed the patient’s follow-up results with their supervisor weekly. They also had the opportunity to share their reflection and obtain feedback from the resource person through the weekly 2 hour plenary session. An electronic form was distributed weekly which can be used by students to report their monitoring progress, challenges, and supports needed. Both peer support and support from the course organizers were continuously provided for all students throughout the course. At the end of the course, students were encouraged to fill students’ satisfaction questionnaires and reflect on their experience in patient monitoring through telemedicine. Each group was also asked to create one education media regarding COVID-19 prevention through health promotion, universal precautions, and COVID-19 vaccination. Details regarding the design and implementation of the course has been previously described elsewhere (27).

### 2.2. Design and data collection

This is a qualitative study using an interpretive phenomenology design (28) to explore students’ self-reflection on their experiences in monitoring patients in self isolation and quarantine for close contacts. This study can be considered as Scholarships of Teaching and Learning (SoTL) where evidence on the values of teaching and learning process in the course was sought systematically and conducted in a robust manner.

Students were asked to reflect on their experiences upon completion of the course through 750–1,000 words self-reflection essays. They were free to choose to write in English or Indonesian. They were also provided with guiding questions: (1) How was your experience and feeling in monitoring patients undergoing COVID-19 self-isolation? (2) Did you face any challenges in monitoring the patients? What were they? Why did it (they) happen to you? (3) What did you learn from those experiences? (4) How do you see your role as a future medical doctor in COVID-19 mitigation? (5) What will you do to prepare yourself as a professional medical doctor in COVID-19 mitigation? Two authors (AF & CH) designed the reflection questions in accordance with self-reflection steps and the research questions. The questions were discussed with other authors for clarity and anticipated responses. Any required amendments were agreed before its administration.

Students submitted their essays through a learning management system and scored by their group tutor using a rubric adapted from O’Sullivan et al. (29) The essays were assessed based on the ability of the students to describe and analyze their experiences and plan their future learning. The current scoring system allowed us to identify such essays, hence high score self-reflective essays (scored above 90 or equal to score 6 of O’Sullivan’s rubric) showed comprehensiveness of the essays. (29–31). The rubric has been used to assess students’ reflective writing in the current setting and has gone through an internal validation process. It is important for the authors in this study to be able to scrutinize meanings of students’ experiences. In addition, gender and student groups’ representativeness were also considered as the sources of variation. All essays were coded with pseudo-initials to avoid selection bias. Prior to the submission, we also asked for students’ consent whose essays were selected.

We ensured saturation of the themes and subthemes by analyzing all included essays meticulously, appropriately used telemedicine and competency conceptual framework to facilitate the coding and development of themes and subthemes and confirmed that there were no new revealed themes and subthemes following the analysis process.

### 2.3. Data analysis

Three researchers (AF, CH, DK) read all written reflections fulfilling the criteria. The code book was developed by the three researchers while reading the documents. The three researchers completed a more detailed independent thematic analysis on three similar self-reflective essays, cross-checked the code book, and discussed and agreed on the revealed themes and subthemes. Other researchers were then involved to provide further feedback on the resulting themes and sub-themes, before the three researchers completed the thematic analysis of the rest of the essays. Subthemes and themes resulted in this study, or any disagreements were discussed by all authors.

All self-reflective essays fulfilling the criteria were analyzed. We attempted to scrutinize units of meanings based on the students’ experiences as described in their essays. Member checking was completed by seeking confirmation from some students’ representatives on the themes and sub themes revealed in the study. The authors were either medical teachers or medical students (AMP) with close involvement in the telemedicine-based course for COVID-19 self-isolation monitoring program. They had adequate understanding of expected competence of medical students in the current setting as well as challenges of telemedicine in patient monitoring as part of students’ learning process in their clinical education and attempted to engage students with public health initiative during the pandemic. The study protocol has been approved by The Research Ethics Committee of Faculty of Medicine Universitas Indonesia No KET-12.15/UN2.F1/ETIK/PPM.00.02/2021.

## 3. Results

A total of 63 reflective essays fulfilled the criteria and were further analyzed in this study. Four main themes emerged from the thematic analysis in relation with COVID-19 isolation monitoring: (1) pearls and pitfalls in communicating with patients through telemedicine, (2) developing professional identity through COVID-19 isolation monitoring, (3) engaging in system-based practice related to COVID-19 isolation monitoring, and (4) experiencing patient-centered care in telemedicine. The themes and subthemes are described in Table 1.

**Table 1:** Themes and subthemes gathered from the study.

Themes	Subthemes	Number of quotes
Pearls and pitfalls in communicating with patients through telemedicine	Communication strategies with patients	43
	Rapport development	30
	Challenges in communication	27
	Empathy development during monitoring	18
	Education of population	10
	Factors supporting communication and education	5
	Role of students in educating patients	5
	Challenges in conducting telemedicine	37
	Benefits of telemedicine	8
	The need of basic knowledge in telemedicine	6
Developing professional identity through COVID-19 isolation monitoring	Sense of responsibility	57
	Sense of purpose	22
	Evidence-based practice and awareness of lifelong learning	18
	The role of *prima facie* in clinical decision making	10
Engaging in system-based practice related to COVID-19 isolation monitoring	Awareness of the importance of comprehensive care and system-based practice	8
	Collaboration in COVID-19 pandemic management	2
Experiencing patient-centered care in Telemedicine	Holistic approach	12
	Trust in doctor-patient relationships	11
	Personalized care	10

### 3.1. Theme 1. Pearls and pitfalls In communicating with patients through telemedicine

Through the module, the students implemented different strategies of developing rapport and empathy during the telemonitoring. For example, they attempted to convey the information as clearly as possible for different characteristics of patients and put further effort to be adaptive in communicating with and educating the patients. This is underlined in one the quotes, as follows:

*I learned that [I could not] apply all methods I knew at their best in practice because of some obstacles. There are limitations, including those from me, … The patients sometimes seemed to ignore the information I gave. They tend to get bored of the monitoring.* (PPH)

The students experienced difficulties in developing rapport, such as patients’ reluctance in engaging in the monitoring process. However, the students learned through their experience and discussion with the tutors on how to overcome those difficulties and made several strategies to facilitate the process. During interactive discussions with students, tutors can offer constructive feedback by giving examples of scenarios that may set off students’ attempts to solve doctor-patient communication issues. The tutor can provide an example of the action or reaction he will take in response to a situation the student encounters.

More importantly, students also learned to develop their empathy toward patients and their family. For example:

*Our experience in this course reflects the reality quite closely that not all patients will be cooperative. We need to keep our patience and try to develop rapport [with the patients]. Those we contacted due to an active tracing program were suspicious of us and refused to be contacted further because they were afraid that we were trying to stigmatize them with COVID-19.* (MEP)

“*Good communication since our initial contact with the patient is the key to a successful monitoring process. We need to choose our words carefully and appropriately, so the patients, who have widely different knowledge and sociocultural backgrounds, can understand us*.” (DAW)

This course provided substantial experience for students to be involved in telemedicine systems which focus on COVID-19 isolation monitoring. Students were aware that interacting with the patients in such systems required their knowledge and readiness in responding to the patients’ concerns and questions appropriately. Some important lessons are described below:

*I attempt to prepare myself well; at least I covered subjects to educate for COVID-19 patients during their self-isolation.* (IA)

Students experienced some challenges in conducting telemonitoring which can be attributed to technology and network limitation, inability to observe and examine patient’s condition accurately, limitation of supportive examination tools and patient’s reluctance and time constraints in reporting their condition every day consistently. Some experiences of the students are elaborated below:

*The family I monitor only has one handphone which is used by the children to participate in online learning in the morning. [Therefore], the patient cannot be contacted during the hours.* (AAS)

*It is hard to observe clinical signs directly during the telemonitoring. I need to educate the patient first on how to measure his/her heart rates, respiratory rate, and dehydration signs if any.* (FN)

### 3.2. Theme 2. Developing professional identity through COVID-19 isolation monitoring

Through students’ involvement with telemonitoring and their ‘direct’ exposure with patients and their family, students reported their increased sense of purpose and responsibility. They could relate to their identity as future medical doctors despite their limited knowledge and experience. For example:

*It was very moving for me when the patient expressed her gratitude because I supported her during the isolation…… I was delighted and I felt like I found my sense of purpose of becoming a medical doctor. I also felt that I got closer to becoming one.* (JoH)

At the same time, they also felt the urge to give more to the community, as some of them intended to participate in the student voluntary program to help the COVID-19 control in the community. Some of students’ realization are as follows:

*I have become more aware that our role as future medical doctors is very much needed [in this pandemic]. I finally signed up as a public relation team member of COVID-19 National Voluntary Program.* (KR)

*The past 4 weeks allowed me to observe that there were a lot of devastating conditions for the patients: they could not see their family [during isolation], their inability to provide income for and to feed their family, and the loss of the family members due to COVID-19. These realities gave me a great motivation to help and to give my best to help more widely during this pandemic.* (AAS)

Concomitantly, two critical features of being professional medical doctors: evidence-based practice and awareness of the importance of lifelong learning, and ethical decision making, were also reported as significant lessons learned which could be obtained by medical students through this telemedicine-based module. The important lessons learned are described below:

*From her question, I knew what aspects to explore further, hence with the interaction I was motivated to keep learning. I attempted to communicate the necessary information to her as needed, and I tried to be honest whenever I could not explain it well and that I needed to confirm further.* (AAR)

*I find that the assessment based on 4 principles of bioethics is very important in dealing with COVID-19 cases. For example, in practicing ‘beneficence’, the patients should be monitored appropriately daily to increase the chance to be healed and to detect warning signs early.* (NBS)

### 3.3. Theme 3. Engaging in system-based practice related to COVID-19 isolation monitoring

Students also shared that the activities they did in the module had exposed them to the current complex healthcare system in regard to COVID-19 pandemic control. This further ignited their realization that they were part of the system. For instance:

*I understand the system to trace close contacts of positive COVID-19 cases better, including its risks and benefits. To me, the current system is quite good to control the pandemic -testing, tracing, treatment. Unfortunately, limited human resources and technical support seem to limit the benefit of the program.* (BA)

Some patients reported confusion of the healthcare system related to COVID-19 pandemic control given inconsistencies across different areas and their lack of information access. As one of the students noted:

*Some patients were very confused by the different policies of COVID-19 pandemic control in different areas. I was reminded by this fact that whenever I become a medical doctor in the future, I will be an inseparable part of the health system.* (AAS)

Through this course, students also realized that for the patient care to be delivered well in the telemonitoring context, multisectoral and interprofessional collaboration are required. Very important notes from the students are highlighted below:

*This module really opened my eyes to at least two things. First, the COVID-19 pandemic control is very complex. Second, this pandemic greatly influenced people’s life biologically, socially, and economically. I think there should have been better coordination between government and other sectors, and there should have been involvement of the people in the attempt.* (PDH)

### 3.4. Theme 4. Experiencing patient-centered care in telemedicine

Given the opportunity to interact with patients quite intensively through the telemedicine platform, students were able to experience and then highlighted the importance of patient-centered and holistic care. Patients’ biopsychosocial factors should always be considered throughout the telemonitoring. Some of the lessons they were highlighted were as follow:

*I should not forget that all patients have their bio-psycho-social condition that needs to be holistically taken care of.* (DO)

Some students also noted that patients with confirmed COVID-19 cases showed more commitment toward the telemonitoring yet with more concerns of their conditions and prognosis, compared to those patients who were still waiting for their examination results or those who were traced actively. The interaction with the patients also contributes to the learning of building trust between doctor-patient. For example:

*……Those with confirmed positive PCR seemed to be more anxious and had a lot of questions, whereas those (close contact) in the self-quarantine seemed to be more relaxed and tend to be hard to contact for telemonitoring.* (SCS)

*I feel that I was trusted by the family since the family also asked for further advice from me.* (ST)

## 4. Discussion

Our study aimed to assess students’ reflection on their experience in conducting virtual monitoring of patients undergoing self-isolation or quarantine for close contacts *via* telemedicine. We utilized an interpretative phenomenology approach toward reflection narratives written by the students at the end of the course to address our research questions. A nationwide multicenter study showed almost half of Indonesian medical students were willing to act as volunteers to help the health authorities mitigate the pandemic, (32) supporting the idea to involve students in pandemic management in a safe yet educational way. The students, under supervision, might help the professionals in conducting telemonitoring, the opportunity would also allow students to gain beneficial skills. Such efforts were shown to be possible and beneficial in similar programs (33, 34). We identified four major aspects learned by the students from their experiences in monitoring patients undergoing self-isolation: communication and education, professionalism and professional identity formation, system-based practice, and patient-centered care.

By involving students first-hand in telemedicine services, students would be able to interact with clients from heterogeneous socioeconomic and educational backgrounds. Since the respondents in our study were mostly early-stage clinical year students with limited patient encounter due to the pandemic, with proper feedback given, this opportunity might be beneficial to build their communication skills and cultural competence (20). Our students also seemed to realize that they cannot simply utilize a “one-size-fits-all” approach and that the best standard practice might not always be possible to be implemented due to resource, system, and socioeconomic constraints.

As previous findings have shown, the stigma and misconceptions related to COVID-19 negatively impact the management (35). Our students were challenged to utilize a personalized approach to each patient they were monitoring, further emphasizing the importance of adaptability and empathy in doctor-patient interaction. With increasing emphasis on cultivating empathetic and communicative future physicians, practice using telemedicine seems to be a good alternative to train students’ communication skills despite the limited face-to-face interaction (33, 36). Albeit done in a different way, empathy and effective communication skills can still be nurtured through virtual interactions. Based on the students’ experience, this study underscores this finding quite strongly. Our study suggests that adequate patient interaction provides a very rich opportunity to learn empathy, communication skills, and mutual decision-making process, despite being conducted virtually. More importantly, students became more aware of patients’ bio-psychosocial background which influenced their adaptation processes in their attempt to provide a patient-centered care approach during the telemonitoring.

A study done in our institution highlighted how the disruptions caused by the pandemic affect the professional identity formation (PIF) of medical students and emphasized the roles of medical schools in supporting the students’ PIF (37). Our current study gives further insight on the beneficial impact of increasing students’ involvement through alternative approaches while giving an adequate amount of support to benefit students’ PIF. Their involvement in this course seemed to reinvigorate their senses of responsibility, purpose, and awareness of the lifelong learning values. This perhaps even accelerates their PIF, with many admitting to feel like they are closer to becoming a professional doctor and showed increased awareness of the real magnitude of health problems posed by the pandemic. They were aware that they need to be more reflective in considering different values and expectations while learning to implement standards of care which significantly show their PIF development (38).

The use of telemedicine, at least in this study context, proved to be satisfactory as students were able to provide care for patients on their expected level of skills and knowledge (as the patients monitored were mostly asymptomatic or mildly symptomatic). This might allow and encourage further participation from the students, which might result in improved decision-making and psychosocial skills (20). This increased participation might explain the beneficial effects observed in our study to the students’ PIF.

Our study suggests that students’ experiences in conducting telemedicine under supervision to monitor COVID-19 patients in self-isolation programs raise their awareness of the importance of system-based practice. They realized that providing care for the patients was beyond doctor-patient relationship as it took place within the healthcare system. In addition to provision of primary health care including in consulting the patients on the emergency conditions, the students should be knowledgeable on the referral system in a certain area during the pandemic. The students’ involvement in this telemedicine-based course can be considered as a strategy to provide a clinical learning environment which enables the system-thinking mindset required for future practice (39). Furthermore, there was also an immense opportunity for students to keep adaptable toward limitation of resources around the patients and their families and overburden of primary health care and hospital care facilities. The deployment of medical students in this program further provides students with a great chance to develop their adaptability amidst uncertainties and creative problem-solving skills (40).

Another important finding from our research is how telemedicine enables delivery of service in line with a patient-centered care framework, as students were shown to collaborate and cooperate with various parties to empower patients, all without interacting directly with the other parties and the patients. In a country with widely different sociocultural backgrounds and collectivist culture like Indonesia, where in-person, face-to-face interaction is still deemed to be irreplaceable, good communication and skills to develop rapport seem to bridge the delivery of care using telemedicine.

Our study provides empirical evidence of the benefits of implementing a telemedicine-based course into the medical curricula as proposed by experts (20, 41), complementing previous research done in similar situations (34). We do realize that despite the seemingly enormous benefits of utilizing telemedicine in medical education, it also requires adequate preparation. Medical schools should ensure proper training is provided for both students and their clinical supervisors as well as ensuring adequate resources are available, including contingency plans for platform/technology failure and should account for disparities to access telemedicine in different regions. In addition, to ensure that the students would learn in the current health system accordingly, advocacy and collaboration of medical schools with health district offices and ministry of health are warranted. Mentorship should also be fostered to ensure proper feedback giving and receiving process as well as promoting engagement with both the student and the patient (42).

One of the strengths of our program is its modular nature with adequate supervision and monitoring throughout the module. Students were also given concise lectures of essential topics by experts and provided with relevant educational resources prior to their telemonitoring activities (27). This, apparently, was beneficial for their practice, as evident in their narratives. Furthermore, the use of telemedicine in medical education as depicted in this study should also consider its limitation that it may not cover all necessary aspects of patient care; consequently, the participation in such a program is considered as complementary clinical learning experience.

Our study has several implications for COVID-19 pandemic control and medical education. First, regarding the pandemic control, our study strengthens the suggestion that encouraging medical students’ participation in the pandemic management by still considering their level of competence is very strategic. The opportunity immerses students with current challenges in clinical practice, nurtures their professional identity formation, and enhances their human competence. Second, telemedicine is likely to persist for future medical practice and should be incorporated into the medical curriculum. This study reports that students required necessary knowledge and skills to take role in the telemonitoring, highlighting the importance of adequate orientation for the medical students prior to their participation in the telemedicine. Third, this study highlights that adaptability should be nurtured among medical students and should be part of the medical curriculum dynamic. In line with relentless effort to fight the pandemic, the adaptations are part of the responsibility of medical students and medical schools to be better equipped for future practice. The telemedicine-based courses should be considered in clinical education to complement direct patient exposure. We believe our findings may inform potential implementation in other contexts where students are enabled to participate in telemedicine-based courses, as shown by the themes from this study.

Our study is not without limitations. We are aware that the potential values of a telemedicine-based course identified in this study are bound with the pandemic situation and our medical curriculum circumstances. We hope to address this limitation by providing a systematic approach in conducting an interpretive phenomenological study and a detailed as well as rich description of the results, hence hopefully it can still be relevant and useful for clinical curriculum development and implementation beyond this pandemic and our curriculum context.

## 5. Conclusion

The Self-Isolation Monitoring for COVID-19 Module was part of an integrative effort involving multiple parties to overcome the immense burden on the nation’s healthcare system during the COVID-19 pandemic. The course intertwined the role of medical school to help the community alleviating the impact of the pandemic through deployment of medical students in self-isolation telemonitoring and the attempt to develop relevant competence of medical students during the pandemic. This telemedicine-based course is proven to provide tremendous learning experiences for the medical students, supporting attempts to develop future-proof medical doctors through various clinical learning experiences.

## Data availability statement

The raw data supporting the conclusions of this article will be made available by the authors, without undue reservation.

## Ethics statement

The studies involving human participants were reviewed and approved by the Research Ethics Committee of Faculty of Medicine Universitas Indonesia No KET-12.15/UN2.F1/ETIK/PPM.00.02/2021. Written informed consent for participation was not required for this study in accordance with the national legislation and the institutional requirements.

## Author contributions

AF, CH, DK, RW, OS, DM, DS, and WW designed the course and the study, while AMP was involved in the implementation of the course. AF led the study. AF, CH, DK, and AMP performed the thematic analysis and translated the participants’ quotes. AF, CH, DK, AMP, and RW drafted the initial manuscript. All authors contributed to the article and approved the submitted version.

## Conflict of interest

The authors declare that the research was conducted in the absence of any commercial or financial relationships that could be construed as a potential conflict of interest.

## Publisher’s note

All claims expressed in this article are solely those of the authors and do not necessarily represent those of their affiliated organizations, or those of the publisher, the editors and the reviewers. Any product that may be evaluated in this article, or claim that may be made by its manufacturer, is not guaranteed or endorsed by the publisher.
